# Association of nasogastric tube or transesophageal echocardiography probe use and head and neck positioning with arytenoid dislocation after endotracheal intubation: a retrospective cohort study

**DOI:** 10.1186/s12871-026-04111-4

**Published:** 2026-07-20

**Authors:** Wei Qi, Guo-rong Wu, Xu-kai Hu, Jun-ping Chen

**Affiliations:** Department of Anesthesiology, Ningbo No.2 Hospital, 41 Xibei Road, Ningbo, 315099 Zhejiang China

**Keywords:** Arytenoid dislocation, Endotracheal intubation, Postoperative hoarseness, Risk factors, Nasogastric tube, Transesophageal echocardiography, Head and neck positioning

## Abstract

**Background:**

Postoperative hoarseness is a common complaint following endotracheal intubation. Arytenoid dislocation (AD) is a structural injury that, if unrecognized, can lead to prolonged voice impairment. This study aimed to identify independent risk factors for AD using routinely collected perioperative variables.

**Methods:**

This retrospective cohort study included adult patients who underwent general anesthesia with endotracheal intubation at a single institution between January 2022 and December 2024. Patients with documented postoperative hoarseness were included. Demographic, surgical, and anesthetic data were collected. Univariate analysis was followed by multivariable logistic regression to identify independent risk factors for AD. To address potential overfitting due to the limited number of AD events (n = 29), we performed a bootstrap internal validation with 1,000 resamples (bias‑corrected accelerated method).

**Results:**

Among 101,204 intubated patients, 141 had documented postoperative hoarseness, and 87 were included after exclusions. Among patients referred for persistent postoperative hoarseness, AD was confirmed in 29 patients (33.3%), with an overall incidence of 0.03%. Multivariable analysis identified nasogastric tube or transesophageal echocardiography probe use (adjusted OR = 13.68; 95% CI: 2.088–89.619; *P* = 0.006) as an independent risk factor for AD. A secondary finding was that intraoperative head/neck positioning showed a positive association in the primary analysis (adjusted OR = 13.34; 95% CI: 1.417–125.612; *P* = 0.024), but bootstrap validation demonstrated instability (BCa 95% CI for β crossed zero), indicating that this finding should be interpreted cautiously. Operative time, surgery type, and anesthetist experience were not significant in the multivariable model.

**Conclusions:**

Esophageal instrumentation requiring either nasogastric tube or transesophageal echocardiography probe appears associated with increased AD risk, while head/neck positioning showed an association that was unstable in bootstrap validation. Although causal inference is limited by the retrospective design and small event number, clinicians should maintain a high index of suspicion for AD in patients with these risk factors to facilitate early diagnosis and timely intervention.

**Supplementary Information:**

The online version contains supplementary material available at https://doi.org/10.1186/s12871-026-04111-4.

## Introduction

Postoperative hoarseness is a common complaint following endotracheal intubation, affecting up to one-third of surgical patients [1, 2]. While most cases resolve spontaneously within days, a subset particularly those with persistent symptoms may harbor arytenoid dislocation (AD), a structural injury that, if unrecognized, can lead to prolonged voice impairment [3, 4]. The estimated incidence of AD is 0.09% [5], though this likely underestimates the true burden given the absence of routine postoperative laryngeal screening. Clinically, AD presents with hoarseness and dysphagia-symptoms indistinguishable from the more common, self-limited laryngeal edema—and is frequently mistaken for vocal fold paralysis, delaying diagnosis and timely intervention [4, 6, 7].

Several perioperative factors have been implicated in AD development [5, 8]. Use of nasogastric tubes (NGT) or transesophageal echocardiography (TEE) probes common in abdominal and cardiac surgeries has been strongly associated with AD, likely due to mechanical pressure on the cricoarytenoid joint [9]. Intraoperative head and neck positioning (extension, flexion, or rotation) may similarly contribute through repetitive tube movement and mucosal contact [10]. Prolonged operative duration (≥ 3 h) has also been identified as a risk factor [9, 11], suggesting a cumulative injury mechanism rather than a single traumatic event. The role of operator experience in AD remains debated. Although not statistically significant in some analyses, residents in training may increase AD risk [8]; first-year residents were more frequently involved in AD cases [10], and trainees with < 2 years' experience using double-lumen tubes had higher postoperative hoarseness rates than senior anesthesiologists [12]. These factors collectively point to a simple but actionable insight: AD risk is determined not only by intubation technique, but also by intraoperative factors that clinicians can monitor.

Recent multidisciplinary guidelines emphasize atraumatic airway techniques, limiting intubation attempts, maintaining cuff pressure within 20–30 cmH₂O, and performing structured postoperative laryngeal assessment to reduce airway‑related morbidity [13, 14]. In this retrospective study, we aimed to evaluate perioperative factors associated with AD and assess the stability of these associations using routinely collected perioperative variables. By clarifying the profile of high-risk patients those with specific surgery types, prolonged operative time, nasogastric tube placement, intraoperative head/neck positioning, or anesthesiologist experience we seek to help clinicians identify patients who warrant closer postoperative voice assessment and earlier otolaryngology referral, ultimately reducing diagnostic delays and improving patient outcomes.

## Methods

### Study design and population

This study was approved by the Ethical Committee of Ningbo No.2 Hospital (Approval No. PJ-NBEY-KY-2025-104-01), which waived the requirement for written informed consent. We retrospectively extracted data for surgical patients who underwent general anesthesia with endotracheal intubation at our institution between January 2022 and December 2024. Patients were excluded if they had pre-existing laryngeal disease, prior head/neck surgery affecting vocal cord function, neurological disorders affecting swallowing or voice, or had incomplete medical records. Patients with postoperative hoarseness were identified through the institutional electronic health record system.

### Data collection

Demographic data obtained included patient age, gender, body mass index (BMI). Surgical data collected included surgical procedure, operating location, operative time, the use of NGT or TEE probes, and intraoperative head/neck positioning. Anesthetic data collected included American Society of Anesthesiologists (ASA) physical status, endotracheal tube size and anesthesiologist experience level.

### Anesthetic management

All patients received standardized general anesthesia according to institutional protocols. Following induction of anesthesia and achievement of adequate neuromuscular blockade, intubations in this cohort were performed using a GlideScope video laryngoscope, direct laryngoscopy was not used. The endotracheal tube cuff was inflated to maintain a pressure of 20–30 cmH₂O per institutional protocol. Tracheal intubations were performed by trainees under the guidance of the consultant anesthetist or by the consultant anesthetist. The tracheal tube, typically 7.0 mm for females and 7.5 mm for males, or 8.0 mm or double-lumen tubes for thoracic surgeries, was gently inserted through the vocal cords. When a stylet was utilized, it was carefully removed once the tube tip passed the glottis. For extubation, the endotracheal tube was removed gently after cuff deflation. Patients who presented with postoperative hoarseness were included in this study.

### Definition of AD

**Fig. 1:**
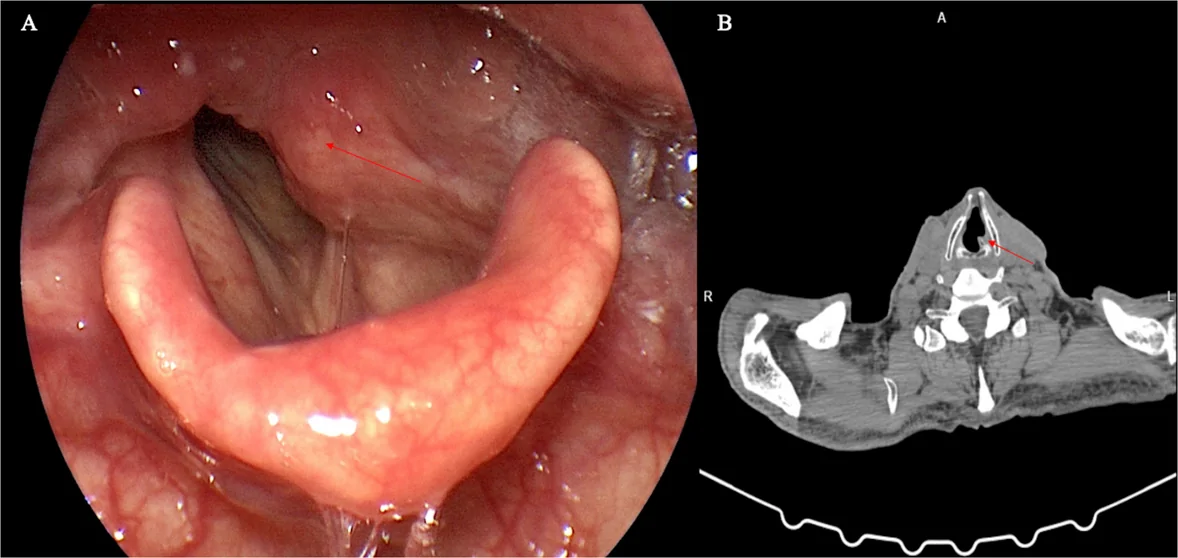
Representative laryngoscopy and CT images of arytenoid dislocation. **A** Fiberoptic laryngoscopy showing left arytenoid dislocation. Note the asymmetric position of the arytenoid cartilages (arrow) and the fixed left vocal fold in the paramedian position. The right vocal fold moves normally during phonation. **B** Axial CT scan of the same patient demonstrating left arytenoid dislocation. The left cricoarytenoid joint space is widened and asymmetrical (arrow) compared with the normal right joint space, consistent with arytenoid dislocation. Radiologic criteria for diagnosis include asymmetry of the cricoarytenoid joint space, obliteration or widening of the joint space, and abnormal anterior or posterior displacement of the arytenoid cartilage relative to the cricoid. *All personally identifiable information has been removed*

The primary outcome was to identify independent risk factors for AD. Patients who presented with hoarseness or pharyngeal discomfort following tracheal intubation underwent evaluation by an otolaryngology specialist. The diagnosis of AD was primarily established via fiberoptic laryngoscopy. Additionally, computed tomography (CT) was utilized to further confirm the diagnosis and assess joint displacement. Representative laryngoscopy and CT images of AD are presented in Fig. 1.

### Statistical analysis

Because this was a retrospective analysis of all eligible patients during the study period, no a priori sample size calculation was performed. Continuous data were presented as median with interquartile range (IQR), and categorical data as frequencies with percentages. For between-group comparisons, normally distributed continuous data were expressed as mean ± standard deviation (SD) and analyzed using Student’s t-test, while non-normally distributed continuous data were expressed as median (IQR) and analyzed using the Mann–Whitney U test. Categorical variables were compared using the chi-square test or Fisher’s exact test, as appropriate. Variables with a *P* < 0.10 in univariate analysis or those considered clinically relevant were entered into a multivariable binary logistic regression model to identify potential risk factors for AD. A *P* < 0.05 was considered statistically significant.

To address potential overfitting due to the limited number of events (n = 29), we performed an additional robustness analysis. Logistic regression was repeated with 1,000 bootstrap resamples using the bias‑corrected accelerated (BCa) method to obtain robust parameter estimates and bias‑corrected confidence intervals. Firth penalized regression was considered; however, because our objective was internal validation of the conventional logistic model used for primary analysis, bootstrap resampling was selected. Statistical analyses were conducted using SPSS version 29.0 (SPSS Inc, Chicago, Illinois, USA).

## Results

**Fig. 2:**
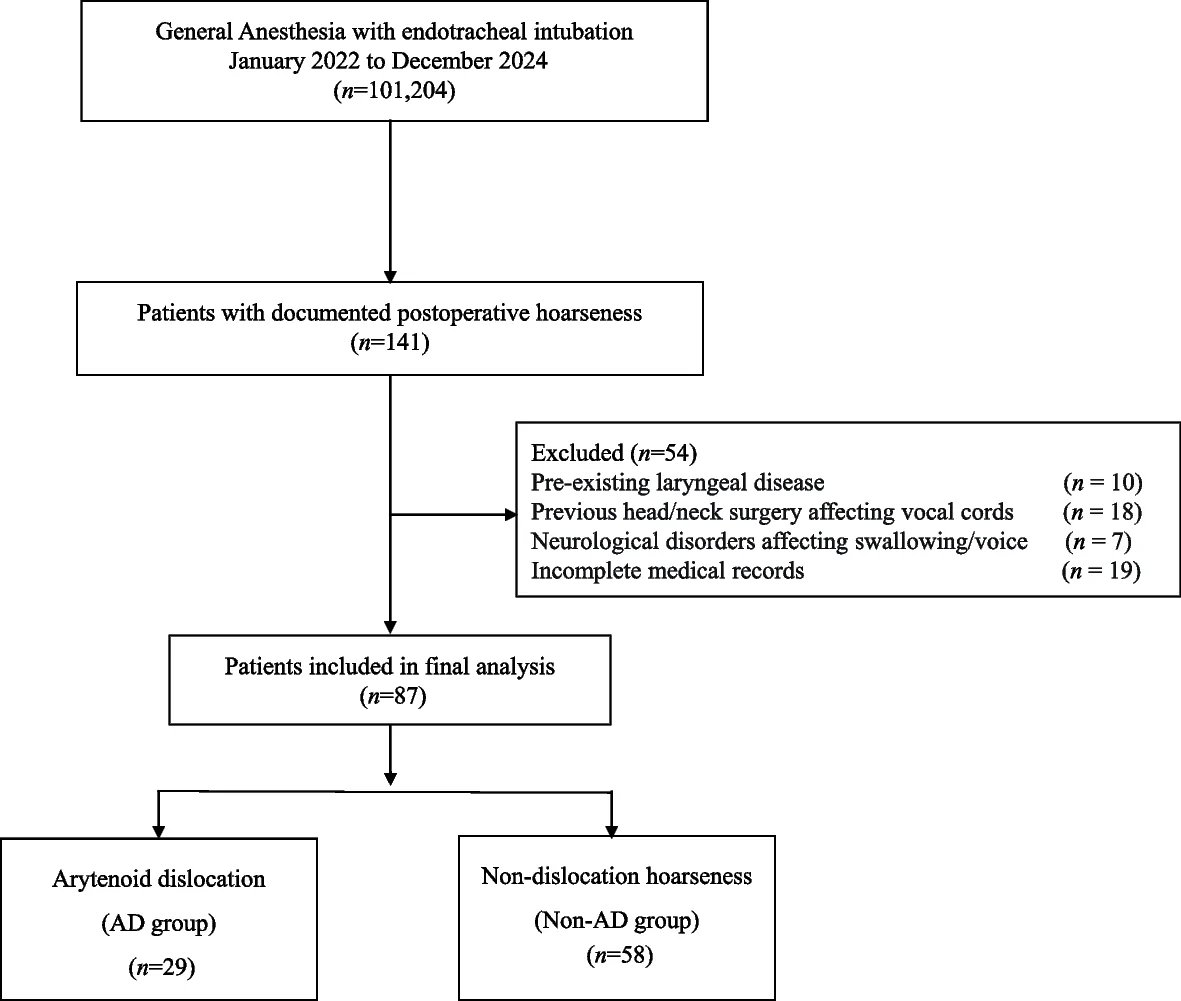
Flow diagram of patient selection and exclusion process

A total of 101,204 patients underwent general anesthesia with endotracheal intubation during the study period. Of these, 141 patients had documented postoperative hoarseness. After applying the exclusion criteria, 87 patients were included in the final analysis (Fig. 2). Among patients referred for persistent postoperative hoarseness, AD was confirmed in 29 patients (33.3%), while the remaining 58 patients (66.7%) comprised the non-dislocation hoarseness group. The overall incidence of AD among all intubated patients was 0.03% (29/101,204).

Patient demographics and clinical characteristics are summarized in Table 1. No significant differences were observed between the AD and non-AD groups in age, female sex, or BMI. The distribution of surgery type did not differ significantly between groups (*P* = 0.10). However, head and neck surgery (13.8% vs. 3.4%) and cardiothoracic surgery (24.1% vs. 13.8%) were more common in the AD group, while abdominal surgery was less common (58.6% vs. 70.7%).

**Table 1:** Univariate analysis of patient characteristics in postoperative hoarseness

Characteristic	**Total (*N* = 87)**	**Non-AD group (*n* = 58)**	**AD group (*n* = 29)**	***P***
Demographics				
Age (years), mean ± SD	**65.29 ± 12.44**	**64.81 ± 12.40**	**66.24 ± 12.68**	**0.308**
Female, n (%)	**47 (54%)**	**30 (51.7%)**	**17 (58.6%)**	**0.353**
BMI (kg/m^2^), mean ± SD	**22.76 ± 3.03**	**22.61 ± 2.96**	**23.04 ± 3.20**	**0.273**
Surgical factors				
Type of surgery, n (%)				**0.10**
Head and neck surgery	**6 (6.9%)**	**2 (3.4%)**	**4 (13.8%)**	
Abdominal surgery	**58 (66.7%)**	**41 (70.7%)**	**17 (58.6%)**	
Cardiothoracic surgery	**15 (17.2%)**	**8 (13.8%)**	**7 (24.1%)**	
Others	**8 (9.2%)**	**7 (12.1%)**	**1 (3.4%)**	
Operative time (min), median (IQR)	**212 (125–275)**	**183 (104–257)**	**272 (145–348)**	**0.004**
Operative time > 3 h, n (%)	**46 (52.9%)**	**26 (44.8%)**	**20 (69.0%)**	**0.028**
NGT/TEE use, n (%)	**55 (63.2%)**	**30 (51.7%)**	**25 (86.2%)**	**0.001**
NGT only	**51 (58.6%)**	**30 (51.7%)**	**21 (72.4%)**	
TEE only	**4 (4.6%)**	**0 (0.0%)**	**4 (13.8%)**	
Head/neck positioning, n (%)	**9 (10.3%)**	**3 (5.2%)**	**6 (20.7%)**	**0.034**
Anesthetic factors				
ASA physical status, n (%)				**0.597**
I ~ II	**66 (75.9%)**	**45 (77.6%)**	**21 (72.4%)**	
III ~ IV	**21 (24.1%)**	**13 (22.4%)**	**8 (27.6%)**	
Endotracheal tube size (mm), n (%)				**0.482**
6.5	**1 (1.1%)**	**1 (1.7%)**	**0 (0.0%)**	
7.0	**31 (35.6%)**	**21 (36.2%)**	**10 (34.5%)**	
7.5	**43 (48.3%)**	**27 (46.6%)**	**15 (51.7%)**	
8.0 or DLT	**13 (14.9%)**	**9 (15.5%)**	**4 (13.8%)**	
Intubation by, n (%)				**0.092**
consultant anesthetist	**35 (40.2%)**	**23 (39.7%)**	**12 (41.4%)**	
first-year residents	**20 (23.0%)**	**13 (22.4%)**	**7 (24.1%)**	
second-year residents	**17 (19.5%)**	**15 (25.9%)**	**2 (6.9%)**	
third-year residents	**15 (17.2%)**	**7 (12.1%)**	**8 (27.6%)**	

*Abbreviations*: *AD* arytenoid dislocation, *ASA* American Society of Anesthesiologists, *BMI* body mass index, *DLT* double-lumen tube, *IQR* interquartile range, *NGT* nasogastric tubes, *SD* standard deviation, *TEE* transesophageal echocardiography

The median operative time was significantly longer in the AD group [272 min (IQR: 145–348)] compared with the non-AD group [183 min (IQR: 104–257)] (*P* = 0.004). The proportion of patients with operative time exceeding 3 h was significantly higher in the AD group (69.0% vs. 44.8%; *P* = 0.028). NGT or TEE probe use was significantly more frequent in the AD group (86.2% vs. 51.7%; *P* = 0.001), as was intraoperative head/neck positioning (20.7% vs. 5.2%; *P* = 0.034). No significant differences were found between groups in ASA physical status, endotracheal tube size distribution, or anesthetist experience level.

Multivariable logistic regression analysis for predictors of AD is presented in Table 2. Variables with *P* < 0.10 in univariate analysis included surgery type, operative time, operative time > 3 h, NGT/TEE use, head/neck positioning, and anesthetist experience. Because the binary variable “operative time > 3 h” was derived from the same continuous variable “operative time (min)” and was highly correlated with it, only the continuous operative time was entered into the multivariable model to avoid collinearity and reduce the number of predictors. Thus, five variables were included in the multivariable logistic regression model. After backward stepwise elimination, NGT or TEE use remained independently associated with AD (adjusted OR = 13.68; 95% CI: 2.088–89.619; *P* = 0.006). Intraoperative head/neck positioning was associated with AD in the primary model (adjusted OR = 13.34; 95% CI: 1.417–125.612; *P* = 0.024). However, as shown in the bootstrap analysis below, this estimate was unstable. In contrast, surgery type, operative time in minutes, and anesthetist experience were not significant in the multivariable model. The Hosmer–Lemeshow test indicated good model fit (χ^2^ = 4.983, *P* = 0.759).

**Table 2:** Multivariable logistic regression analysis of independent risk factors for arytenoid dislocation

Risk factor	β	SE	Wald χ^2^	*P*	Adjusted OR	95% CI
Type of surgery	0.347	0.336	1.070	0.301	1.415	(0.733–2.731)
Operative time (min)	0.003	0.003	0.908	0.341	1.003	(0.997–1.008)
NGT/TEE use	2.616	0.959	7.440	0.006	13.680	(2.088–89.619)
Head/neck positioning	2.591	1.144	5.129	0.024	13.342	(1.417–125.612)^†^
Anesthetist experience	−0.638	0.554	1.327	0.249	0.528	(0.178–1.565)
Constant	−3.110	1.333	5.440	0.020	0.045	

*Abbreviations*: *β* regression coefficient, *CI* confidence interval, *NGT* nasogastric tubes, *OR* odds ratio, *SE* standard error, *TEE* transesophageal echocardiography

^†^Bootstrap analysis showed that the BCa 95% CI for β crossed zero for head/neck positioning, indicating instability; this estimate should be interpreted cautiously

To assess the robustness of our findings given the small number of AD events, we performed a bootstrap internal validation with 1,000 resamples; the results are shown in Supplementary Table S1. The bootstrap analysis confirmed that NGT/TEE use remained a significant predictor (bootstrap *P* = 0.002; BCa 95% CI for β: 0.547–72.567). In contrast, bootstrap analysis demonstrated instability of the estimate for head/neck positioning: although the bootstrap *P* value was 0.004, the BCa 95% confidence interval for β crossed zero (–0.930 to 41.763). The wide confidence interval for head/neck positioning (1.417–125.612) reflects the small number of exposed patients (*n* = 9) and the limited number of AD events in this subgroup (*n* = 6), consistent with sparse-data bias. Due to zero TEE probe use in the non‑AD group (0/58 vs. 4/29 in the AD group), separate logistic regression for TEE alone was not feasible because of complete separation.

## Discussion

In this retrospective study, we systematically analyzed the clinical characteristics and risk factors for AD in patients presenting with hoarseness following endotracheal intubation. The overall incidence of AD in our cohort was 0.03%, which is lower than the pooled incidence of 0.093% reported in recent meta-analyses [5]. This lower incidence likely reflects underdiagnosis due to selective referral of patients with persistent hoarseness [15], as AD may spontaneously recover or be misdiagnosed as recurrent laryngeal nerve paralysis [16]. However, a critical finding was that among the subpopulation presenting with postoperative hoarseness, AD was confirmed in 33.3% of cases. This high proportion underscores the clinical imperative to maintain a high index of suspicion for AD in patients who develop hoarseness after surgery, rather than dismissing it as transient vocal fold edema. Early diagnosis is critical, as prompt diagnosis and treatment can prevent complications, improve prognosis, and avoid cricoarytenoid joint ankylosis [17].

A key finding of our study is the strong association between NGT or TEE probe use and the occurrence of AD. This aligns with previous research identifying NGT use as a strong independent risk factor [9]. In a propensity-matched analysis, the use of a NGT showed the strongest association with AD, with an odds ratio of 23.9 [9]. The proposed mechanism is the mechanical irritation and destabilization of the cricoarytenoid joint cavity caused by the indwelling tube [18]. Specifically, an external force affecting the cricoarytenoid joint can result in loosening of the joint capsule, and congested synovium can be squeezed into the joint cavity, resulting in edema and affecting the joint's stability [7]. Furthermore, procedures involving esophageal instrumentation, such as the insertion of a NGT or TEE, have been highlighted as factors that heighten the risk of AD [5, 19].

Our primary analysis suggested an association between intraoperative head/neck positioning and AD. However, bootstrap internal validation indicated that this association was unstable, as the BCa 95% CI for β crossed zero. The extremely wide confidence interval for the odds ratio (1.417–125.612) is a classic sign of sparse-data bias, which occurs when the number of events in one exposure category is very small. In our cohort, only 9 patients had documented head/neck positioning, of whom 6 developed AD. Therefore, this finding should not be interpreted as evidence of an independent risk factor, but rather as a hypothesis‑generating observation that requires confirmation in larger, adequately powered studies.

Nevertheless, a hypothesis-generating observation does not preclude biological plausibility, and the existing mechanistic literature offers plausible explanations that merit consideration. Head-neck movement during surgery increases the risk of AD due to altered patient positioning [10, 20]. It has been reported that the tip of the endotracheal tube in the trachea is displaced significantly with head-neck movement, up to a median of 5.0 cm [20]. This displacement of the cuffed tracheal tube, along with head positioning, may cause inadvertent trauma to the cricoarytenoid joint [10]. Another possible explanation is that the displaced cuffed tracheal tube may have exerted prolonged pressure against the arytenoid cartilage and thereby inadvertently dislocated it during surgery [21]. This risk is particularly pertinent in surgeries such as anterior neck or brain surgery where specific head and neck positioning is a prerequisite for better surgical exposure [10]. While these mechanistic observations are consistent with our primary analysis, the statistical instability of the estimate means they remain speculative and require validation in future studies specifically designed to test this hypothesis.

Our findings support a cumulative microtrauma model rather than a single intubation-related event. During surgery, head and neck movement can displace the endotracheal tube tip by up to 5.0 cm, causing repeated shearing forces on the cricoarytenoid joint [20]. An indwelling NGT or TEE probe further destabilizes the joint capsule through mechanical irritation. Esophageal instrumentation requiring either NGT or TEE appears associated with increased AD risk (This mechanism is consistent with previous studies showing that NGT use carries an odds ratio of 23.9 for AD [9] and that the combined NGT/TEE variable is independently associated with AD (adjusted OR = 13.68)). Prolonged operative time (> 3 h) has also been identified as a risk factor [9], likely because longer surgery increases cumulative exposure to these mechanical stressors. Additionally, patients undergoing abdominal surgery appear to be at higher risk [9], which may be mediated by more frequent NGT placement and longer duration. Even extubation, if performed with an incompletely deflated cuff, may exert traction on the arytenoid cartilage [4]. In this framework, AD risk accumulates throughout the intraoperative period, which explains why our multivariable model retained only persistent intraoperative exposures (NGT/TEE use and head/neck positioning) and not transient events such as intubation difficulty or operator experience. This concept aligns with contemporary airway management guidelines that emphasize limiting intubation attempts to no more than three, maintaining cuff pressure within 20–30 cmH₂O, and performing routine postoperative airway assessment [13, 14]. The guidelines also recommend video laryngoscopy as the first-choice device to reduce airway trauma and advocate preoperative airway evaluation and multidisciplinary team involvement [14]. Early treatment, such as closed reduction within 13–26 days, can achieve favorable outcomes [22].

Our results underscore the importance of minimizing unnecessary esophageal instrumentation and stabilizing head/neck position during surgery, as even minor repetitive trauma may contribute to dislocation. Nevertheless, the evidence for head/neck positioning was less robust, given its instability in bootstrap validation, and should be interpreted cautiously.

Although univariate analysis in this study demonstrated a significant association between prolonged operative time and AD (*P* = 0.004), this variable did not retain statistical significance in the multivariate regression model (*P* = 0.341). This finding suggests that operative time may function as a confounding variable rather than an independent risk factor. It is plausible that longer surgeries are associated with greater surgical complexity and a higher probability of utilizing specific instruments, such as NGT or TEE probes. Previous studies have identified that procedures involving esophageal instrumentation significantly heighten the risk of AD [5, 9, 16]. The mechanical irritation and destabilization of the cricoarytenoid joint caused by these indwelling tubes provide a more direct pathophysiological mechanism for dislocation [23]. Therefore, in the multivariate model, the inclusion of variables like NGT use may have absorbed the variance attributed to operative time, revealing that the duration of surgery itself is likely a surrogate marker for the intensity of airway manipulation and instrument usage rather than a direct cause of AD [5].

Interestingly, several factors traditionally considered potential risks [3, 10, 24] did not emerge as independent predictors in our multivariate analysis. Beyond the prolonged operative time, specific surgeries like abdominal procedures have been linked to AD [9], our multivariate analysis did not find surgery type to be an independent risk factor. This indicates that the occurrence of AD may be more directly related to specific intraoperative manipulations, such as traction or damage to the vagus nerve, or the use of tubes, rather than the surgical category itself.

Regarding demographic factors, our study found no significant association between gender and AD. This contrasts with some reports suggesting female sex is a risk factor [10], but aligns with other studies [8, 9, 11] that found demographic characteristics such as gender did not show statistical significance. Furthermore, contrary to previous reports indicating that less experienced anesthetists increase AD risk [8, 12], we did not find the experience of anesthetists to be an independent predictor. This aligns with a retrospective case–control study that found no correlation between the anesthesiologist's experience and AD occurrence [9]. Additionally, AD can occur even with the use of video laryngoscopes [25], suggesting that the mechanism of injury is complex and not solely dependent on operator experience. AD results from multifactorial, cumulative intraoperative factors rather than a single intubation-related event.

While these findings support a multifactorial model of AD, several limitations warrant consideration. First, as a retrospective single‑center study with only 29 AD events, our findings may not be generalizable. The true incidence of AD (0.03%) is likely underestimated because patients with mild symptoms may not have been referred for laryngoscopy. Although bootstrap resampling (1,000 resamples, BCa intervals) helped address small‑sample bias, the wide confidence intervals and the fact that the CI for head/neck positioning crossed zero indicate that the exact magnitudes of these associations are uncertain. Although bootstrap validation improved internal stability, the small events‑per‑variable ratio may still have resulted in overestimation of effect sizes. The large odds ratios and extremely wide confidence intervals suggest sparse‑data bias, and these estimates should therefore be interpreted as signals of association rather than precise risk magnitudes. This is particularly relevant for head/neck positioning, which was present in only 9 patients (6 AD cases), contributing to the instability of its estimate.

Second, the absence of a standardized postoperative voice screening protocol introduced selection bias. Hoarseness was documented at clinician discretion without a fixed threshold for otolaryngology referral, leading to systematic exclusion of patients with mild or transient hoarseness. This bias likely underestimates the true incidence of AD (our 0.03% vs. the meta‑analytic 0.09% [5]) and may overestimate its proportion among documented hoarseness cases (33.3%).

Third, the definition of “head/neck positioning” in this study did not distinguish between extension, flexion, or rotation, nor did it quantify the duration or degree of positioning. These factors may differentially affect the stress exerted on the cricoarytenoid joint. Furthermore, the combined “NGT/TEE use” variable, although supported by a common pathophysiological mechanism, may obscure differential risk magnitudes between the two devices. Due to zero TEE exposure in the control group, device‑specific multivariable analysis was not possible. Prospective studies with standardized intraoperative positioning records and device‑specific data collections are warranted to clarify these associations.

Fourth, several airway‑related variables were unavailable (Cormack‑Lehane grade, intubation attempts, use of stylets or bougies, extubation technique). Although all intubations used GlideScope video laryngoscopy with protocol‑driven cuff pressure (20–30 cmH₂O), residual confounding cannot be excluded. Future prospective studies should standardize documentation of airway management, intraoperative positioning, and laryngeal screening to validate our findings.

In conclusion, AD is a rare but non-negligible complication following endotracheal intubation. Despite the limitations inherent to a retrospective design and a small number of events, our study suggests that esophageal instrumentation requiring either NGT or TEE is associated with increased AD risk. Head/neck positioning showed a positive association in the primary analysis, but this finding was unstable in bootstrap validation and requires confirmation in larger studies. For patients undergoing procedures involving NGT/TEE placement or requiring intraoperative head/neck positioning, clinicians should maintain a low threshold for postoperative voice assessment and otolaryngology referral. Prospective multicenter studies are needed to confirm these findings and to evaluate preventive strategies.

## Supplementary Information

Supplementary Material 1.

## Data Availability

The datasets used and analyzed during the current study are available from the corresponding author on reasonable request.
