# Supplementary material for: Association of nasogastric tube or transesophageal echocardiography probe use and head and neck positioning with arytenoid dislocation after endotracheal intubation: a retrospective cohort study
Source: BMC Anesthesiol. 2026 Jul 20;26:617. doi: 10.1186/s12871-026-04111-4 (PMC13663125; doi:10.1186/s12871-026-04111-4)
Supplement: Supplementary file 1 [file 12871_2026_4111_MOESM1_ESM.docx]

**Supplementary Table S1. Logistic regression with bootstrap (1000 resamples, BCa 95% confidence intervals) for risk factors of arytenoid dislocation.** For each predictor, the unstandardized coefficient (β), bootstrap bias, bootstrap standard error, bootstrap *P* value (two‑sided), and the 95% BCa confidence interval for β are shown.

| Risk factor | β | Bootstrap Bias | Bootstrap SE | Bootstrap *P* (2‑tailed) | BCa 95% CI for β | OR | 95% BCa CI for OR |
| --- | --- | --- | --- | --- | --- | --- | --- |
| Type of surgery | 0.347 | 0.195 | 1.732 | 0.219 | (-8.094, 17.557) | 1.41 | (0.000, 4.2×10⁷) |
| Operative time (min) | 0.003 | 0.000 | 0.003 | 0.382 | (-0.004, 0.009) | 1.003 | (0.996, 1.009) |
| NGT/TEE use | 2.616 | 3.241 | 8.827 | 0.002 | (0.547, 72.567) | 13.69 | (1.73, 1.9×10³¹) |
| Head/neck positioning | 2.591 | 3.267 | 8.142 | 0.004 | (-0.930, 41.763) | 13.36 | (0.39, 1.4×10¹⁸) |
| Anesthetist experience | -0.638 | -0.039 | 0.706 | 0.289 | (-2.104, 0.562) | 0.53 | （0.12, 1.75） |
| Constant | -3.110 | -3.402 | 9.411 | 0.003 | (-6.537, -2.469) | 0.045 | (0.001, 0.085) |

Abbreviations: β, regression coefficient; BCa, bias‑corrected accelerated; CI, confidence interval; NGT, nasogastric tubes; OR, odds ratio; SE, standard error; TEE, transesophageal echocardiography.
